# Alveolar ridge augmentation in Oral and Maxillofacial Surgery: a study on current practices, patient management and innovations in Germany

**DOI:** 10.1186/s40729-025-00619-5

**Published:** 2025-04-16

**Authors:** Andreas Pabst, Jörg Wiegner, Matthias Schneider, Nils Weyer, Alexander Bartella, Max Heiland, Philipp Becker, Alexander-N. Zeller

**Affiliations:** 1 Department of Oral and Maxillofacial Surgery, University Medical Center Mainz, Augustusplatz 2, 55131 Mainz, Germany; 2 Department of Oral and Maxillofacial Surgery, German Armed Forces Central Hospital, Rübenacherstr. 170, 56072 Koblenz, Germany; 3 Private Practice for Oral and Maxillofacial Surgery, Saalstr. 35, 07318 Saalfeld, Germany; 4 Private Practice for Oral and Maxillofacial Surgery, Dr.-Külz-Ring 15, 01067 Dresden, Germany; 5 Private Practice for Oral and Maxillofacial Surgery, Fabrikstr. 10/1, 73728 Esslingen a.N., Germany; 6 Private Practice for Oral and Maxillofacial Surgery, Detmolder Str. 530, 33699 Bielefeld, Germany; 7 Department of Oral and Maxillofacial Surgery, University Medical Center Leipzig, Liebigstr. 12, 04103 Leipzig, Germany; 8 Department of Oral and Maxillofacial Surgery, Charité – Universitätsmedizin Berlin, Corporate Member of Freie Universität Berlin and Humboldt-Universität zu Berlin, Augustenburger Platz 1, 13353 Berlin, Germany; 9 Private Practice for Oral and Maxillofacial Surgery, Theaterstr. 61, 52062 Aachen, Germany; 10 Department of Oral and Maxillofacial Surgery, Hannover Medical School, Carl-Neuberg-Str. 1, 30625 Hannover, Germany

**Keywords:** Oral- and Maxillofacial Surgery, Alveolar ridge augmentation, Bone substitute, Survey, Dental implants

## Abstract

**Introduction:**

This study identifies current practices, patient management concepts and innovations in alveolar ridge augmentation (ARA) in Oral and Maxillofacial Surgery (OMFS) in Germany.

**Material and methods:**

A survey with a dynamic online questionnaire with up to 40 questions was designed to collect general and specific data on ARA, such as case numbers, imaging, surgical techniques, (bio-)materials, and case management in OMFS. After internal and external validation, 1863 OMF surgeons within the German Association for Oral and Maxillofacial Surgery (DGMKG) were invited via email to participate. Data management was anonymized and descriptively.

**Results:**

324 OMF surgeons participated in the study (response rate 17.39%). Most participants (60.8%) work in private practices without inpatient care. 62.03% of participants insert > 200 implants, and 28.70% perform > 200 ARA annually. About 30.86% also provide implant-based prosthetic restorations. Cone-beam computed tomography (CBCT) is the imaging method most preferred by 87.74% of participants. The most common ARA techniques are external and internal sinus lift (SL) and bone block augmentation (97.31%, 90.57%, and 73.4%, respectively). Intraoral harvested autogenous bone grafts (ABG) are most commonly used for ARA (96.63%). The oblique line is the participants’ preferred donor site for ABG (93.27%). ABG and xenogeneic bone substitutes are the most frequently used graft combinations (72.73%). Platelet-rich fibrin (PRF) is used by 58.59% of participants for ARA, mostly in SL procedures (76.44%). PRF is the most common substitute used to biofunctionalize biomaterials (48.16%). Oral antibiotics are used by 86.40% pre-/intraoperatively and by 88.97% postoperatively for ARA. Most participants believe the surgical technique (94.49%) and the surgeon's experience (92.28%) are the most critical factors for ARA success. 46.32% of participants aim to perform ARA within the skeletal envelope.

**Conclusion:**

The findings highlight current practices, patient management, and innovations in ARA in OMFS in Germany. They show standard practices and numerous variations in several aspects.

**Clinical relevance:**

Surgical technique, experience, patient health and compliance are relevant ARA success factors. This underlies the importance of extended surgical training and careful patient selection.

**Supplementary Information:**

The online version contains supplementary material available at 10.1186/s40729-025-00619-5.

## Introduction

Alveolar ridge augmentation (ARA) is essential to oral regeneration and implantology. It enables implant placement in jaw areas with severe bone loss, which may reach approximately 11–22% vertically and 29–63% horizontally approximately six months after tooth loss [1]. In such cases, ARA can facilitate implant placement and implant-supported prosthetic restorations, improving patients' oral health-related quality of life [2]. Overall, ARA may be required in approximately 50% of cases before or during implantation, although this rate depends on various factors [3]. ARA includes many procedures, from standard sinus lift and guided bone regeneration (GBR) to more complex approaches, including bone blocks and shells harvested from the iliac crest. ARA has continuously advanced over recent years, with innovative techniques and biomaterials significantly expanding its potential. These include, for example, CAD/CAM technologies, navigation systems, and resorbable magnesium scaffolds [4–6]. Additionally, the biofunctionalization of biomaterials, such as bone substitutes (BS) and matrices, using autologous platelet concentrates (e.g., platelet-rich fibrin, PRF) or hyaluronic acid (HA) has gained significance in clinical practice [7–9]. In this context, the background of biofunctionalization is to approximate the properties of processed and acellular biomaterials to those of autologous grafts [10]. Furthermore, high-resolution imaging modalities, such as cone-beam computed tomography (CBCT), have further enhanced implant placement, which can, in turn, influence ARA and vice versa [11]. Unfortunately, many aspects of ARA remain unclear and require further investigation, including optimal surgical techniques, the use of grafts and (bio-)materials for specific alveolar ridge defects, and pre-, peri-, and postoperative case management. Further key focus areas include pain management, antibiotic use, complication management, and identifying success factors. The skeletal envelope and its role in ARA and alveolar relining using xenogeneic BS are also not clarified in detail [12, 13]. Additionally, trends such as diameter- and length-reduced implants, minimally invasive surgical techniques, and guided surgery are emerging, which may render ARA unnecessary in some cases [14]. This information is crucial for identifying gaps in current knowledge and emphasizing the need for further research and guideline development to refine and expand the indications for ARA. Next, detailed information about current practices in ARA is missing. This study analyzed current practices, patient management and innovations in ARA in Oral and Maxillofacial Surgery (OMFS) in Germany.

## Material and methods

A dynamic online questionnaire with up to 40 questions was designed to collect general and specific data and information on ARA in OMFS in Germany. General data included the participants’ professional and occupational profiles and annual case numbers in implantology and ARA. Specific data on ARA covered, among others, factors such as imaging methods, surgical techniques, ARA procedures, grafts and (bio-)materials, ABG donor sites, pre-, peri- and postoperative case management, complications and complication management, relative and absolute contraindications, and success factors for ARA.

The dynamic online questionnaire included single- and multiple-choice questions and partially an option for free-text answers. Participants could leave out or omit one or more questions within the survey. Specific answers automatically triggered follow-up questions for some questions, which were not presented if a different answer was given. The percentage values (%) in the answers represent the proportion of participants who selected a specific answer to a question relative to the total number of participants who answered that question. Due to the possibility of choosing multiple answers in the multiple-choice questions, the sum of percentage values can exceed 100%. Supplemental material 1 presents an overview of the questionnaire, including the questions and the respective answer options for each question.

The authors independently performed internal validation of the questionnaire. Experienced surgeons and recognized experts in implantology and ARA performed external validation of the questionnaire separately from one another. The steps were carried out in succession. After internal and external validation, the questionnaire was implemented into SurveyMonkey’s^®^ online platform (SurveyMonkey Inc., San Mateo, California, USA; de.surveymonkey.com). This was followed by an additional internal evaluation by the authors to ensure the functionality of the questionnaire in SurveyMonkey^®^.

The survey link, along with a cover letter to the participants, was emailed to 2078 OMF surgeons within the German Association for Oral and Maxillofacial Surgery (Deutsche Gesellschaft für Mund-, Kiefer- und Gesichtschirurgie e.V., DGMKG) who were included in the DGMKG head office’s email distribution list. Of these, 169 emails were undeliverable, and an additional 46 email addresses had opted out of SurveyMonkey^®^ surveys. In total, the survey link successfully reached 1863 participants. Three reminders were sent to increase participation after one, five, and seven weeks. The survey was closed after 10 weeks. Data management was conducted anonymously and descriptively, ensuring that personal associations between the data and the participants were excluded.

The data was transferred to Excel^®^ (Microsoft Corporation, Redmond, WA, USA). Figures were generated using GraphPad Prism^®^ (GraphPad Software, San Diego, CA, USA). Language and grammar checks were conducted using ChatGPT 4.0^®^ (OpenAI, San Francisco, CA, USA) and Grammarly^®^ (Grammarly Inc., San Francisco, CA, USA).

## Results

### General data

In total, 324 OMF surgeons participated in the study, resulting in a response rate of 17.39%. Most participants (60.8%, 197/324) reported working in private practices without inpatient care facilities (no dedicated or associated beds). A smaller proportion (18.83%, 61/324) were affiliated with private practices offering inpatient care with dedicated or associated beds. Other settings included OMFS departments of hospitals (8.64%, 28/324), university hospitals (8.02%, 26/324), general dental practices (2.16%, 7/324), and oral surgery practices (1.54%, 5/324). Seventy-one out of 324 participants (21.91%) reported placing more than 500 implants yearly. Additionally, 18.21% (59/324) inserted 201–300 implants annually, while 12.35% (40/324) placed 101–200 implants. The distribution of implant placement was more evenly spread across other categories: 11.11% (36/324) placed 301–400 implants, 10.80% (35/324) inserted 401–500 implants, and 10.19% (33/324) placed 51–100 implants annually. Some participants reported lower activity levels, with 8.02% (26/324) placing 21–50 implants and 7.41% (24/324) inserting 0–20 implants annually. Concerning the number of ARA, the most prominent groups performed 51–100 ARA (19.75%, 64/324) and 101–200 ARA (19.44%, 63/324) yearly. A smaller proportion (16.98%, 55/324) reported performing 21–50 procedures yearly, while 15.12% (49/324) conducted fewer than 20 ARA annually. Further, 12.04% (39/324) of participants performed 201–300 ARA yearly. Some participants reported higher levels of activity: 5.86% (19/324) conducted 301–400 ARA, 3.70% (12/324) performed 401–500, and 7.10% (23/324) reported conducting more than 500 ARA annually. Concerning implant-supported prosthetic treatments alongside augmentative and implantological procedures, most participants (69.14%, 224/324) stated that they do not carry out such prosthetic treatments. A smaller number (26.85%, 87/324) reported performing them occasionally, while only 4.01% (13/324) stated that they always conduct prosthetic treatments as part of their practice. There were various reasons for not performing implant-supported prosthetic treatments in addition to implantological and augmentative procedures. Most participants cited the reason for working in a purely surgical referral practice (79.09%, 174/220). Lack of experience or expertise was the second most frequent reason (24.09%, 53/220), followed closely by lack of interest (22.73%, 50/220) for this field. Other reasons included the procedure to be performed by another colleague in the practice or clinic (16.36%, 36/220) and low demand from referring dentists or patients (13.64%, 30/220). Some participants (3.18%, 7/220) answered no financial benefit as a deterrent.

### Imaging, virtual planning, guided surgery, and navigation

**Fig. 1:**
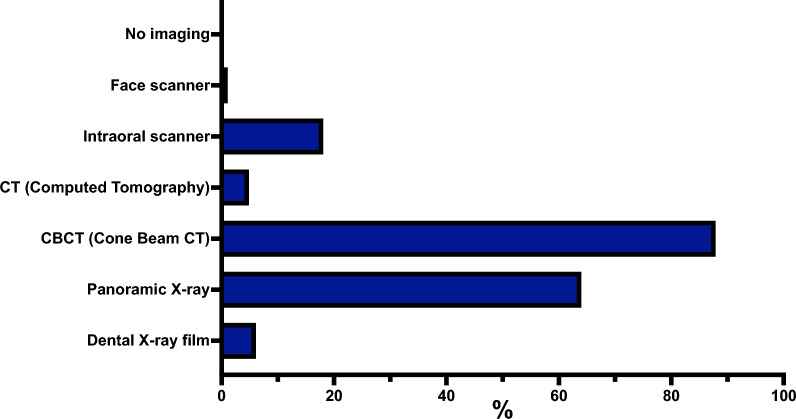
Overview of the preferred imaging modalities for planning implantations and ARA. Data is presented as percentages (%), showing the proportion of participants who elected each imaging modality relative to the total number of participants who answered the question (Supplemental material 2)

Figure 1 overviews the preferred imaging modalities for planning implantations and ARA. Concerning using virtual planning software (e.g., coDiagnostiX^©^, SMOP^©^, etc.) in augmentative and implantological procedures, 19.18% of participants (61/318) reported using virtual planning in all cases. Most participants (45.28%, 144/318) indicated using such software only in selected cases. None of the participants (0.00%, 0/318) reported using virtual planning for ARA and a planned later implantation or exclusively for implantation following ARA. A significant proportion, 35.53% (113/318), stated that they do not use virtual planning software. Various reasons for not using virtual planning in augmentative and implantological procedures were found. The most frequently cited reason was the perception of a lack of benefit, reported by 54.05% (60/111) participants. Additionally, 38.74% (43/111) stated no demand from referring clinicians or patients. A poor cost–benefit ratio was reported by 36.94% (41/111), while high hardware and software costs were cited by 21.62% (24/111). Lack of experience or expertise was mentioned by 22.52% (25/111), and insufficient personnel resources by 9.01% of participants (10/111). Only 3.60% (4/111) of participants indicated that previous negative experiences deterred them from using virtual planning. Regarding using guided surgery techniques (e.g., guided surgery with templates or infrared-based navigation) in augmentative and implantological procedures, 9.87% of participants (31/314) reported using guided surgery in all cases. In comparison, 56.37% (177/314) indicated employing it only in selected cases. None of the participants (0.00%, 0/314) used guided surgery for ARA and planned later implantation or exclusively for implantation following ARA. A significant number of participants, 33.76% (106/314), stated that they do not use guided surgery. Regarding the types of guided surgery used for augmentative and implantological procedures, all users (100.00%, 207/207) indicated using guided surgery with templates. A small of these participants, 0.97% (2/207), reported using infrared-based navigation, while 2.90% (6/207) selected “other”, non-defined reasons. Various reasons were identified for not using guided surgery for augmentative and implantological procedures. The most frequently cited reason was the perception of a lack of benefit, reported by 58.49% (62/106) of participants. A poor cost–benefit ratio was mentioned by 45.28% (48/106), followed by high hardware and software costs, reported by 28.30% (30/106). Additionally, 31.13% (33/106) indicated a lack of demand from referring clinicians or patients. Lack of experience or expertise was reported by 15.09% (16/106), and previous negative experiences by 11.32% (12/106). Insufficient personnel resources were cited by 6.60% of participants (7/106).

### Augmentation techniques, grafts, bone substitutes, and combinations

**Fig. 2:**
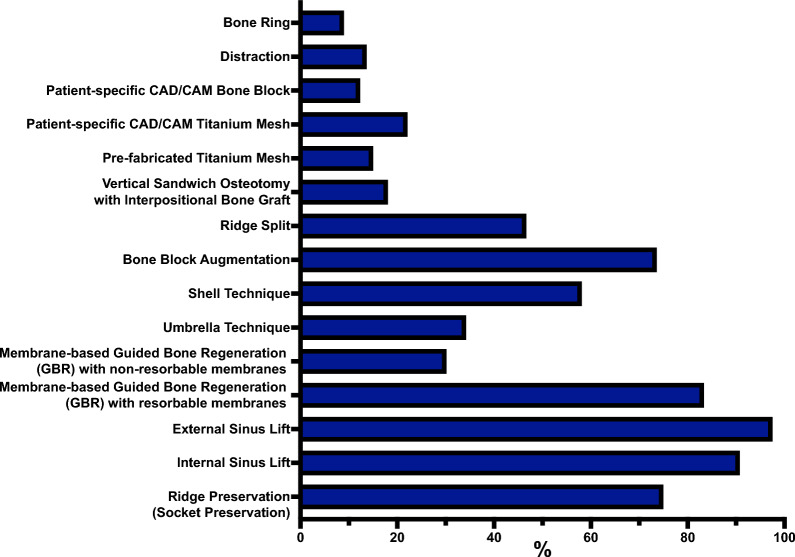
Overview of the ARA techniques performed by the participants. Data is presented as percentages (%), showing the proportion of participants who selected each ARA technique relative to the total number of participants who answered the question (Supplemental material 2)

Figure 2 overviews the ARA techniques performed by the participants. The most commonly utilized site for harvesting autologous bone was the oblique line, reported by 93.27% (277/297) participants. The implantation site was used by 71.38% (212/297), followed by the zygomaticoalveolar crest, reported by 55.89% (166/297). Other frequently mentioned sites included the mental region (33.67%, 100/297) and the iliac crest (33.33%, 99/297). Less commonly utilized donor sites were the external table of the skull (3.37%, 10/297) and the tibial head (3.03%, 9/297). The most frequently used instrument for harvesting and processing autologous bone was the bone scraper, reported by 78.45% (233/297) of participants. Piezo surgery was also widely used, with 71.72% (213/297) indicating its use, followed by trephine drills, reported by 55.22% (164/297). Bone mills were used by 49.16% (146/297) and saws by 41.41% (123/297). Cutting discs were employed by 39.39% (117/297), while bone filters attached to suction systems were the least frequently used, reported by 26.94% of participants (80/297). Table 1 describes the kind and frequency of complications seen when harvesting autologous bone. Intraorally harvested autologous bone was most commonly used, reported by 96.63% (287/297) participants regarding the types of bone and BS used for ARA. Xenogeneic BS (e.g., bovine) was the second most frequently used, stated by 76.43% (227/297). Synthetic BS (e.g., hydroxyapatite, tricalcium phosphate) was utilized by 40.40% (120/297), closely followed by allogeneic bone, reported by 40.07% (119/297). Extraorally harvested autologous bone was used by 32.32% of participants (96/297). Regarding the combinations of bone and BS used for ARA, autologous bone with xenogeneic BS was most commonly used, reported by 72.73% (216/297) participants. This was followed by autologous bone combined with synthetic BS, used by 36.70% (109/297), and autologous bone with allogeneic bone, reported by 29.63% (88/297). Less frequently used combinations included allogeneic bone with xenogeneic BS (10.10%, 30/297) and allogeneic bone with synthetic BS (4.04%, 12/297). The least frequently used combination was xenogeneic BS with synthetic BS, reported by 1.35% of participants (4/297).

**Table 1:** Overview of the kind and frequency of complications participants see when harvesting autologous bone

	Very Frequent (%) / n	Frequent (%) / n	Occasionally (%) / n	Rarely (%) / n	Very Rarely (%) / n	Total Responded (n)
Pain	8.45% / 25	28.04% / 83	41.89% / 124	15.54% / 46	6.08% / 18	296
Swelling	15.82% / 47	58.25% / 173	19.87% / 59	4.38% / 13	1.68% / 5	297
Infection/Abscess	0.00% / 0	0.00% / 0	9.31% / 27	36.90% / 107	53.79% / 156	290
Postoperative bleeding	0.00% / 0	0.35% / 1	7.96% / 23	38.75% / 112	52.94% / 153	289
Wound healing disorder	0.00% / 0	0.35% / 1	15.97% / 46	42.71% / 123	40.97% / 118	288
Temporary nerve damage	0.00% / 0	0.00% / 0	8.45% / 24	20.42% / 58	71.13% / 202	284
Permanent nerve damage	0.00% / 0	0.00% / 0	0.72% / 2	2.16% / 6	97.12% / 270	278

Complications are categorized into five groups: Very Frequent, Frequent, Occasionally, Rarely, and Very Rarely, with corresponding percentages and absolute counts (n). The "Total Responded" column indicates the total number of participants who reported each symptom. Data is presented as percentages (%) and absolute numbers (n)

### Membranes and platelet-rich fibrin

**Fig. 3:**
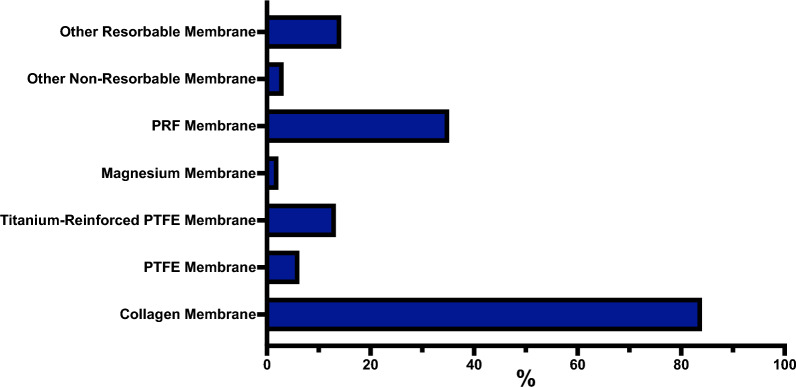
Overview of the membrane types preferred for GBR by the participants. Data is presented as percentages (%), showing the proportion of participants who selected each membrane type relative to the total number of participants who answered the question (Supplemental material 2)

Concerning the types of membranes used for ARA, collagen membranes were the most frequently used, reported by 88.89% (264/297) of participants. PRF membranes were also widely used by 48.48% of participants (144/297). Titan-reinforced polytetrafluorethylene (PTFE) membranes were reported by 22.90% (68/297), while PTFE membranes were used by 13.13% (39/297). Other resorbable membranes employed 18.86% (56/297), whereas other non-resorbable membranes were used by 5.39% (16/297). Magnesium membranes were the least frequently used, reported by 3.03% (9/297) participants. Figure 3 overviews the membrane types preferred for GBR by the participants. Regarding the use of PRF for ARA, 29.97% of respondents (89/297) reported using PRF in most cases, while 28.62% (85/297) indicated its use in selected cases. However, 41.41% (123/297) said they do not use PRF for ARA. Table 2 overviews the indications areas for PRF reported by participants. Regarding the reasons for not using PRF for ARA, most participants (48.36%, 59/122) answered a perception of a lack of benefit. Lack of experience or expertise was mentioned by 29.51% (36/122), while 27.87% (34/122) indicated no demand from referring clinicians or patients. A poor cost–benefit ratio was reported by 24.59% (30/122), and 22.95% (28/122) cited high equipment costs as a barrier. Insufficient personnel resources were noted by 20.49% (25/122), and only 2.46% of participants (3/122) reported previous negative experiences with PRF.

**Table 2:** Overview of the indications areas for PRF reported by participants

Indications	Percentage (%)	Responses (n)
Ridge Preservation (Socket Preservation)	64.37%	112
Sinus lift without KEM/autologous bone	27.01%	47
Sinus lift with KEM/autologous bone (e.g., covering the Schneiderian membrane)	76.44%	133
Sticky bone	70.11%	122
Biologization of KEM	56.32%	98
Biologization of membranes	50.57%	88
Biologization of implant surfaces	28.16%	49
PRF membrane over defect before wound closure	74.14%	129
Soft tissue corrections	27.59%	48
Total Participants		174

Each indication area is described in the left column, while the right columns display the percentage of participants who selected each option and the absolute number of responses (n)

### Selection criteria for augmentation techniques, grafts, bone substitutes, and membranes

**Fig. 4:**
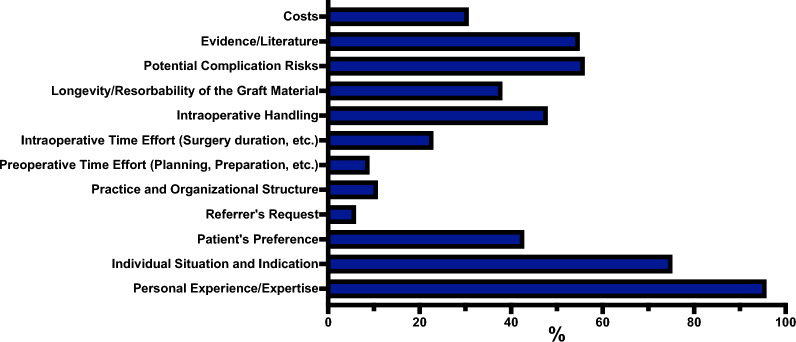
Overview of the factors influencing the ARA choice of bone grafts and BS. Data is presented as percentages (%), showing the proportion of participants who selected each factor relative to the total number of participants who answered the question (Supplemental material 2)

**Fig. 5:**
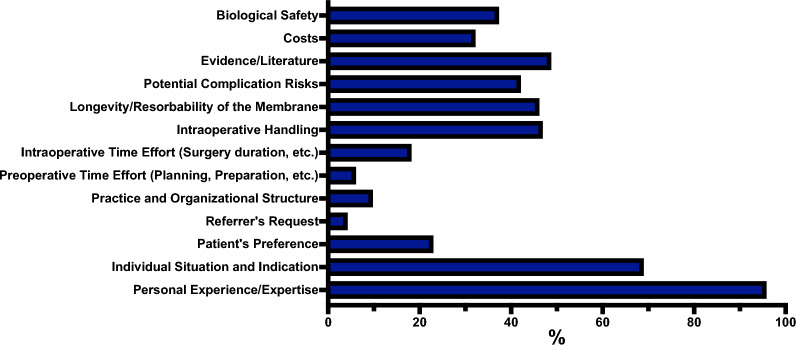
Overview of the factors influencing the ARA choice of membranes. Data is presented as percentages (%), showing the proportion of participants who selected each factor relative to the total number of participants who answered the question (Supplemental material 2)

The most decisive factor influencing the choice of ARA technique was personal experience or expertise, reported by 97.06% (264/272) participants. Potential complication risks were also highly influential, mentioned by 61.03% (166/272), followed by evidence or literature, reported by 55.15% (150/272). Intraoperative time requirements were considered important by 32.72% (89/272), and patient preferences were cited by 31.99% (87/272). Costs were a determining factor for 32.35% (88/272), while preoperative time requirements influenced 18.01% (49/272). Additional factors included economic reasons (19.49%, 53/272), practice and organizational structure (15.07%, 41/272), and referrer’s preferences (8.09%, 22/272). Figures 4 and 5 overview factors influencing the ARA choice of bone grafts, BS, and membranes.

### Pre-, intra- and postoperative augmentation management strategies

**Fig. 6:**
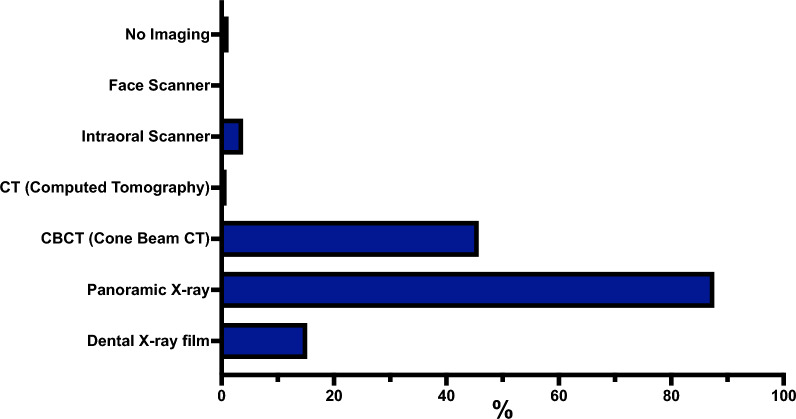
Overview of the preferred imaging modalities after ARA. Data is presented as percentages (%), showing the proportion of participants who selected each imaging modality relative to the total number of participants who answered the question (Supplemental material 2)

The most frequently employed pre- and intraoperative ARA management strategy was oral antibiotic therapy, reported by 86.40% (235/272) participants. CHX rinses were also widely used by 76.84% of participants (209/272). Smoking cessation was implemented by 58.46% (159/272), and optimization of oral hygiene was emphasized by 52.57% (143/272). Professional dental cleaning was used by 41.91% (114/272), while optimization of patient compliance was cited by 40.07% (109/272). Glucocorticoids (e.g., dexamethasone) were administered by 30.88% (84/272), and preemptive analgesia (e.g., ibuprofen) by 29.04% (79/272). Intravenous antibiotic therapy was the least frequently used strategy, reported by 17.28% (47/272). Regarding postoperative ARA management strategies, the most commonly used strategy was oral antibiotic therapy, reported by 88.97% (242/272) participants. Cooling was the second most common measure, employed by 83.46% (227/272). Smoking cessation was implemented by 76.10% (207/272), and preventing prostheses in the augmentation area was emphasized by 78.68% (214/272). Physical rest or prohibition of sports was reported by 73.16% (199/272). As-needed pain medication was used by 70.22% (191/272), while a predefined pain medication regimen was employed by 33.82% (92/272). Chlorhexidine (CHX) rinses were used by 59.56% (162/272), and soft relining of tissue-borne prostheses was mentioned by 30.51% (83/272). Less frequently used strategies included arnica (8.46%, 23/272), bromelain (7.72%, 21/272), and kinesio tape (2.21%, 6/272). Intravenous antibiotics were the least frequently reported strategy, used by 6.99% of participants (19/272). Table 3 overviews the kind and frequency of complications participants see after ARA. Regarding factors influencing the long-term success of ARA, most participants (94.49%, 257/272) cited the surgical technique, followed closely by the experience or expertise of the operator, indicated by 92.28% (251/272). Patient health and compliance were also considered crucial, cited by 81.25% (221/272), while oral hygiene was emphasized by 77.57% (211/272). Smoking status was reported as an important factor by 83.46% (227/272), and the quality of materials was mentioned by 62.87% (171/272). Medications and comorbidities of the patient were considered significant by 62.50% (170/272), and aftercare was highlighted by 51.84% of participants (141/272). Figure 6 overviews the preferred imaging modalities after ARA. Tables 4 and 5 overview relative and absolute contraindications for ARA stated by the participants. Regarding vitamin D testing in patients before ARA, most participants (83.82%, 228/272) reported not utilizing vitamin D testing. Of those who do, 10.29% (28/272) indicated testing via blood samples sent to an external laboratory, while 6.25% (17/272) perform the testing directly in their practice.

**Table 3:** Overview of the kind and frequency of complications participants see after ARA

	Very Frequent (%) / n	Frequent (%) / n	Occasionally (%) / n	Rarely (%) / n	Very Rarely (%) / n	Total Responded (n)
Infections	0.00% / 0	0.00% / 0	20.45% / 55	44.61% / 120	34.94% / 94	269
Abscesses	0.00% / 0	0.00% / 0	2.26% / 6	21.43% / 57	76.32% / 203	266
Postoperative bleeding	0.00% / 0	0.00% / 0	8.99% / 24	31.84% / 85	59.18% / 158	267
Membrane exposure	0.37% / 1	3.75% / 10	33.33% / 89	32.58% / 87	29.96% / 80	267
Dehiscence	0.37% / 1	2.61% / 7	38.81% / 104	34.70% / 93	23.51% / 63	268
Wound healing disorders	0.00% / 0	1.12% / 3	30.97% / 83	46.64% / 125	21.27% / 57	268
Insufficient augmentation volume during the course	0.00% / 0	0.75% / 2	36.09% / 96	39.85% / 106	23.31% / 62	266
Augmentation loss	0.00% / 0	0.37% / 1	5.97% / 16	36.19% / 97	57.46% / 154	268

Complications are categorized into five groups: Very Frequent, Frequent, Occasionally, Rarely, and Very Rarely, with corresponding percentages and absolute counts (n). The "Total (n)" column indicates the total number of participants who reported each complication. Data is presented as percentages (%) and absolute numbers (n)

**Table 4:** Overview of relative contraindications for ARA stated by the participants

Relative Risk Factors	Percentage (%)	Responses (n)
Well-controlled diabetes mellitus	7.35%	20
Poorly-controlled diabetes mellitus	68.38%	186
Radiation in the augmentation area within the last year	58.46%	159
Radiation in the augmentation area, not within the last year	50.37%	137
Use of proton pump inhibitors	5.88%	16
Antiresorptive for benign underlying disease	58.46%	159
Antiresorptive (high potency) for malignant underlying disease	57.72%	157
Smoking	58.46%	159
Poor oral hygiene	77.21%	210
Poor compliance	73.53%	200
Mucosa-supported prostheses in the augmentation area	27.21%	74
Total participants		272

Relative contraindications are listed alongside the percentage of respondents who reported it and the absolute number of responses (n). Data is presented as percentages (%) and absolute numbers (n)

**Table 5:** Overview of absolute contraindications for ARA stated by the participants

Absolute Risk Factors	Percentage (%)	Responses (n)
Well-controlled diabetes mellitus	0.00%	0
Poorly-controlled diabetes mellitus	37.13%	101
Radiation in the augmentation area within the last year	83.09%	226
Radiation in the augmentation area, not within the last year	30.51%	83
Use of proton pump inhibitors	0.37%	1
Antiresorptive for benign underlying disease	13.24%	36
Antiresorptive (high potency) for malignant underlying disease	77.21%	210
Smoking	14.34%	39
Poor oral hygiene	29.41%	80
Poor compliance	38.60%	105
Mucosa-supported prostheses in the augmentation area	8.82%	24
Total participants		272

Absolute contraindications are listed alongside the percentage of respondents who reported it and the absolute number of responses (n). Data is presented as percentages (%) and absolute numbers (n)

### Advancements and challenges in augmentations

Regarding promising innovative developments in ARA, the most frequently cited innovation was the use of stem cells, reported by 31.62% (86/272) participants. This was followed by bioprinting of bone and BS, mentioned by 26.84% (73/272), and advancements in CAD/CAM technology, indicated by 26.47% (72/272). Similarly, 26.47% (72/272) of participants found innovative resorbable biomaterials (e.g., magnesium screws) promising. New synthetic biomaterials were highlighted by 22.79% (62/272), while intraoperative navigation was seen as promising by 16.91% (46/272). Notably, 31.25% (85/272) of participants indicated that none of the mentioned developments were promising. The most frequently cited challenge in applying biomaterials (BS and membranes) in practice was cost, reported by 66.91% (182/272) participants. Long-term stability of ARA was highlighted by 43.38% (118/272), and concerns regarding behavior in cases of peri-implantitis were noted by 41.91% (114/272). Biological safety (e.g., cell debris, bacteria, viruses, prions) was considered a challenge by 31.62% (86/272). Handling issues were reported by 28.31% (77/272), while time requirements and market overview/product diversity were each mentioned by 26.47% (72/272) and 29.78% (81/272), respectively. Storage requirements posed a challenge for 23.16% (63/272), while documentation was cited by 19.85% (54/272). Acceptance by patients was reported as a concern by 24.63% (67/272), and personnel resources and availability were challenges for 9.19% (25/272) and 8.82% (24/272) participants, respectively. Regarding their sources of information about biomaterials, continuing education was the most frequently mentioned source, cited by 89.34% (243/272) participants. Conferences were also a popular source, indicated by 79.78% (217/272). Professional journals were used by 75.37% (205/272), and scientific literature by 71.32% (194/272). Colleagues were considered a source of information by 66.91% (182/272), while Internet sources were used by 40.07% (109/272). Representatives from companies were the least frequently mentioned source, reported by 41.91% of participants (114/272).

### General principles, skeletal envelope, and biomaterial biologization in augmentation

Regarding the agreement with general statements about ARA, the most agreed-upon statement was “As little as possible, as much as necessary,” supported by 70.22% (191/272) participants. The use of autologous bone and soft tissue as the gold standard was endorsed by 54.78% (149/272), while 45.96% (125/272) considered biomaterials a good alternative to autologous grafts. Statements such as avoiding ARA whenever possible and preferring short and diameter-reduced implants over ARA were agreed upon by 24.26% (66/272) and 21.69% (59/272), respectively. 20.59% of participants (56/272) supported augmenting generously and preventively when necessary. Regarding the significance of the skeletal envelope (also known as the bony envelope) for ARA, most participants (56.25%, 153/272) stated to augment situationally within and outside the skeletal envelope. Another 46.32% (126/272) reported attempting to augment strictly within the skeletal envelope, while 12.50% (34/272) indicated augmenting outside the skeletal envelope. A smaller proportion, 13.60% (37/272), stated that the skeletal envelope holds no significance for them, and only 1.10% of participants (3/272) reported not considering the skeletal envelope during augmentations. The most frequently used suture material was non-resorbable monofilament sutures, selected by 61.40% (167/272) participants. Resorbable braided sutures were used by 22.06% (60/272), while 20.59% (56/272) preferred non-resorbable braided sutures. Resorbable monofilament sutures were chosen by 18.75% (51/272). Tissue adhesives (e.g., cyanoacrylate) were rarely used, reported by only 0.74% of participants (2/272). PRF was the most frequently used method for biologization of biomaterials (BS and membranes), reported by 48.16% (131/272) participants, followed by autologous blood, used by 42.28% (115/272). Platelet-rich plasma (PRP) was employed by 24.63% (67/272), while enamel matrix proteins (e.g., Emdogain©) were used by 19.49% (53/272). Growth factors (e.g., BMP-2) were rarely used, reported by 1.10% (3/272). 20.59% (56/272) of participants indicated that they do not use any methods of biologization.

## Discussion

This study analyzed current practices, patient management, and innovations in ARA in OMFS in Germany. Most participants reported performing many implant placements and ARA annually, showing its established role in OMFS in Germany. About 70% of participants do not provide implant-supported prosthetic restorations, reflecting the surgical focus in implantology and oral rehabilitation in OMFS in Germany.

Most participants (87.74%) favored CBCT for preoperative implant and ARA planning. CBCT enables precise surgical planning by analyzing alveolar ridge dimensions, defect sizes, and critical anatomical structures (e.g., inferior alveolar nerve) in detail [15–17]. It further allows for virtual planning and static and dynamic guided surgery, improving implant placement accuracy and reducing complications [18–21]. Next, it enables dynamic intraoperative navigation that could further improve implant placement, enhance safety measures, and increase ARA efficiency, particularly in sinus lifts [22]. Raabe et al. reported a nearly 20% increase in computer-assisted implant surgeries over the past 20 years [14], which could result from the mentioned advantages. In this context, a previous study demonstrated that implantology is the main application area for printed guides in OMFS in Germany [23]. Nearly two-thirds of participants in the current study stated that they use virtual planning. The reasons mentioned for not using virtual planning were a perceived lack of benefit, no demand from patients or referring colleagues, and an unfavorable cost–benefit ratio. Next, approximately 65% of participants reported using guided surgery in all or selected cases, suggesting a visible match between virtual planning and implementing guided surgery. This seems to be a comprehensible step to implement virtual planning 1:1 intraoperatively on the patient. Even without surgical guides, CBCT and virtual implant planning may offer advantages over conventional imaging [24]. The primary reasons cited by participants for not using guided surgery were a perceived absence of benefits and an unfavorable cost–benefit ratio. A reason may be that even guided surgery implantation can have deviations: axial from 0–5.64°, apical from 0.01–3.2 mm, and coronal from 0.01–1.6 mm [25]. Next, guided surgery may be associated with further challenges, such as in the edentulous jaw, where precise and stable positioning of the guide can be challenging and which can lead to deviations [26]. Such limitations can be improved using transitional implants to fix surgical guides and provisional prostheses [27]. The in-house production of surgical guides may reach a cost reduction, while only about 20% of the OMFS hospitals and private practices in Germany have a 3D printer [23]. Barriers to virtual planning and guided surgery, such as limited benefits and costs, should be addressed through education and cost-effective solutions to enhance their adoption and integrate these technologies more widely. Further implementations may include deep learning models to segment CBCT scans before ARA and implant placement [28] and artificial intelligence, e.g., in treatment planning [29]. Participants’ preferred ARA techniques were sinus lift (external and internal) and GBR with resorbable membranes. Sinus lifts are commonly used due to limited alternatives for the lateral upper jaw and their reputation as safe procedures with low complication rates and predictable long-term success rates [30, 31]. Implant survival rates in cases with and without membrane perforation during sinus lift procedures are about 97% [32]. GBR is a well-documented and widely used method for ARA in alveolar ridge defects, with sufficient evidence and high long-term implant success rates [33]. Resorbable collagen matrices were the participants’ most popular for ARA and GBR. The reasons may be their ease of use and the fact that they don’t require removal. A recent network meta-analysis by Calciolari et al. found that complications, such as dehiscence and membrane exposure, are higher when using cross-linked membranes by trend [34]. This is confirmed in other studies reporting membrane exposure rates 30% higher for cross-linked compared to non-cross-linked membranes [35]. This may give evidence that non-cross-linked collagen matrices with a natural collagen structure could be superior. The current study found a trend of avoiding complex ARA techniques such as sandwich osteotomy, titanium meshes, CAD/CAM bone blocks, distraction osteogenesis, and bone rings. This may be due to the many alternatives, costs, and higher risk of complications. Nevertheless, even the mentioned techniques have their areas of indication with sufficient clinical results, such as vertical sandwich osteotomy in the atrophic posterior maxilla [36, 37]. Intraorally harvested ABG (96.63%), particularly from the oblique line, and xenogeneic BS (76.43%) were the most commonly used grafts and BS for ARA. The combination of ABG and xenogeneic BS is frequently practiced (72.73%). These materials may synergistically promote bone regeneration through their osteoconductive and -inductive properties, resorption protection (e.g., relining), and reduced donor site morbidity [12, 38, 39]. PRF is used by nearly 60% of participants for multiple indications, including sinus lift with grafts and BS, wound healing, sticky bone (e.g., for GBR), and ridge preservation, demonstrating its broad range of applications. PRF was reported to improve wound healing and reduce pain [40, 41]. There is further evidence that leukocyte (L)-PRF may positively influence bone regeneration [41]. Its popularity among the participants may also be due to its simple production and its 100% autologous character [42]. The results indicated a clear trend in the use of antibiotics before and after ARA. A meta-analysis supports that preoperative oral antibiotics (amoxicillin) in ARA can reduce early implant failure in one-stage procedures and lower bacterial load of graft material for one- and two-stage procedures [43]. Other strategies, such as preventive pain and swelling management and using a structured plan for postoperative pain medication, appear to be less commonly implemented. This is surprising since the current study reported pain and swelling as commonly occurring complications. Preemptive analgesia (e.g., etoricoxib) has been shown to reduce pain and improve the quality of life following third molar extraction [44]. Comparable outcomes were observed for preemptive ibuprofen use in third molar extractions, resulting in decreased postoperative pain [45]. Another study demonstrated that 4 mg dexamethasone could reduce postoperative swelling in third molar extractions [46]. Overall, the benefits of preemptive analgesia seem without controversy. The results of this study showed that the success of ARA heavily depends on surgical technique and the surgeon's experience, according to the participants, highlighting the need for thorough training and education. The reliance on personal experience suggests a lack of standardized guidelines, which could be resolved with consensus-based protocols. Next, early implementation of implantology in surgical training must be performed. A recent study has shown that nearly 45% of OMFS residents intend to specialize in implantology. However, the study also revealed that the initial interest in implantology at the beginning of residency significantly declined as the training progressed. This trend may suggest a potential underrepresentation of implantology and ARA during OMFS residency programs in Germany [47]. The skeletal envelope concept is also controversially discussed. About 45% of participants augment within it, while 56% do so outside it. There's no consistent definition of the skeletal envelope in the literature. One possible definition may be the original contour of the bony alveolar ridge or the soft tissues before tooth loss and bone resorption [48]. The hypothesis is that augmentation outside the envelope may be more difficult with higher resorption and complications than inside. Therefore, studies should further specify the definition and significance of the skeletal envelope for implantology and ARA. Future advancements in stem cell therapies, bioprinting, CAD/CAM technologies, and innovative biomaterials could revolutionize ARA with customizable, patient-specific solutions. Additionally, researching minimally invasive techniques and alternative implant designs may decrease the necessity for ARA. A future trend will be avoiding ARA, e.g., by shorter and diameter-reduced implants. Narrow-diameter implants are emerging as a viable option to regular-diameter implants with bone augmentation, especially in the anterior maxilla and preventing ARA [49]. This is supported by most participants’ statement to augment “as little as possible, as much as necessary”.

## Conclusions

Numerous variations in current practices and patient management were found, highlighting ARA techniques’ dynamic and evolving nature. The causes may be multifactorial and variable. There may be a need for further guideline development. ARA success factors, according to participants, include surgical techniques, experience, patient health and compliance. This may underline the importance of extended surgical training and careful patient selection. Further studies should be conducted to evaluate whether innovations, such as advanced imaging, navigation tools, and graft and biomaterial biofunctionalization, may improve ARA success rates and, thus, patient satisfaction. Regarding biomaterials, challenges such as costs, long-term ARA stability, and materials’ behavior in cases of periimplantitis could be addressed to enable broader adoption of biomaterials in clinical practice.

## Supplementary Information

Supplementary material 1. Overview of the questionnaire.

Supplementary material 2. Corresponding data to figures 1–6.

## Data Availability

Data are available upon reasonable request.
