# Supplementary material for: Alveolar ridge augmentation in Oral and Maxillofacial Surgery: a study on current practices, patient management and innovations in Germany
Source: Int J Implant Dent. 2025 Apr 16;11:31. doi: 10.1186/s40729-025-00619-5 (PMC12003255; doi:10.1186/s40729-025-00619-5)
Supplement: Supplementary file 1 — Supplementary material 1. Overview of the questionnaire. [file 40729_2025_619_MOESM1_ESM.docx]

**In which facility do you work as an oral and maxillofacial surgeon for implantology?**

- Referral practice for oral and maxillofacial surgery without inpatient care (no dedicated beds/guest beds)

- Referral practice for oral and maxillofacial surgery with inpatient care (dedicated beds/guest beds)

- Oral surgery practice

- General dental practice

- Hospital (oral and maxillofacial surgery department)

- University clinic

**How many implants do you insert on average per year?**

- 0–20

- 21–50

- 51–100

- 101–200

- 201–300

- 301–400

- 401–500

- >500

**How many augmentations do you perform on average per year?**

- 0–20

- 21–50

- 51–100

- 101–200

- 201–300

- 301–400

- 401–500

- >500

**Do you also perform implant-supported prosthetic restorations in addition to augmentation and implantology procedures?**

- YES, always

- YES, occasionally

- NO

**Why do you not perform implant-supported prosthetic restorations in addition to augmentation and implantology procedures? (Multiple answers possible)**

- Lack of experience/expertise

- Lack of interest

- No financial benefit

- Performed by another colleague in the practice/clinic

- No demand from referrals and/or patients

- Work exclusively in a surgical referral practice

**What imaging do you prefer for planning implantations or augmentations? (Multiple answers possible)**

- Dental film

- Panoramic radiograph

- CBCT

- CT

- Intraoral scanner

- Face scanner

- No imaging

**Do you use virtual planning (e.g., coDiagnostiX©, SMOP, etc.) for augmentation and implantology procedures?**

- YES, in all cases

- YES, in selected cases

- YES, for both augmentation and planned subsequent implantation

- YES, only for implantation after augmentation

- NO

**Why do you not use virtual planning for your augmentation and implantology procedures? (Multiple answers possible)**

- Lack of experience/expertise

- Poor experiences

- No benefit

- No demand from referrals and/or patients

- Lack of personnel resources

- Poor cost-benefit ratio

- High cost of hardware and software

**Do you use "Guided Surgery" (template-based or infrared-based navigation) for your augmentation and implantology procedures? (Multiple answers possible)**

- YES, in all cases

- YES, in selected cases

- YES, during both augmentation and planned subsequent implantation

- YES, only during implantation after augmentation

- NO

**What type of "Guided Surgery" do you use for your augmentation and implantology procedures? (Multiple answers possible)**

- Template-based guided surgery

- Infrared-based navigation

- Other (please specify)

**Why do you not use Guided Surgery? (Multiple answers possible)**

- Lack of experience/expertise

- Poor experiences

- No benefit

- No demand from referrals and/or patients

- Lack of personnel resources

- Poor cost-benefit ratio

- High cost of hardware and software

**What types of augmentations do you perform? (Multiple answers possible)**

- Ridge preservation (socket preservation)

- Internal sinus lift

- External sinus lift

- Membrane-based guided bone regeneration (GBR)

- Umbrella technique

- Shell technique

- Bone block augmentation

- Ridge split

- Vertical sandwich osteotomy with interposition graft

- Prefabricated titanium mesh

- Patient-specific CAD/CAM titanium mesh

- Patient-specific CAD/CAM bone block

- Distraction

- Bone ring technique

**What donor sites do you use for harvesting autologous bone? (Multiple answers possible)**

- Zygomatic-alveolar crest

- Retromolar/oblique line region

- Mental region

- Iliac crest

- Outer cortical plate

- Tibial head

- Implantation site

**What instruments do you use for harvesting and processing autologous bone? (Multiple answers possible)**

- Saw

- Cutting disc

- Piezo surgery

- Trephine drill

- Bone scraper

- Bone mill

- Bone filter on the suction device

**What complications related to bone harvesting do you generally observe during or after the procedure? (Multiple answers possible)**

- Pain

- Swelling

- Infection/abscess

- Postoperative bleeding

- Wound healing disturbance

- Temporary nerve damage

- Permanent nerve damage

**What bone or bone substitute materials (BSMs) do you use in augmentations? (Multiple answers possible)**

- Autologous bone (intraoral donor site)

- Autologous bone (extraoral donor site)

- Allogenic bone

- Xenogenic BSM (e.g., bovine)

- Synthetic BSM (e.g., hydroxyapatite, tricalcium phosphate)

**What combinations of bone or BSMs do you use in augmentations? (Multiple answers possible)**

- Autologous bone + allogenic bone

- Autologous bone + xenogenic BSM

- Autologous bone + synthetic BSM

- Allogenic bone + xenogenic BSM

- Allogenic bone + synthetic BSM

- Xenogenic BSM + synthetic BSM

**What membranes do you use in augmentations? (Multiple answers possible)**

- Collagen membrane

- PTFE membrane

- Titanium-reinforced PTFE membrane

- Magnesium membrane

- PRF membrane

- Other non-resorbable membrane

- Other resorbable membrane

**What membranes do you prefer for GBR? (Multiple answers possible)**

- Collagen membrane

- PTFE membrane

- Titanium-reinforced PTFE membrane

- Magnesium membrane

- PRF membrane

- Other non-resorbable membrane

- Other resorbable membrane

**Do you use platelet-rich fibrin (PRF) in augmentations?**

- YES, in most cases

- YES, in selected cases

- NO

**In what ways do you use PRF? (Multiple answers possible)**

- Ridge preservation (socket preservation)

- Sinus lift without BSM/autologous bone

- Sinus lift with BSM/autologous bone (e.g., covering Schneiderian membrane)

- Sticky bone

- Biologizing BSM

- Biologizing membranes

- Biologizing implant surfaces

- PRF membrane over defect before wound closure

- Soft tissue corrections

**Why do you not use PRF? (Multiple answers possible)**

- Lack of experience/expertise

- Poor experiences

- No benefit

- No demand from referrals and/or patients

- Lack of personnel resources

- Poor cost-benefit ratio

- High cost of equipment

**What factors are decisive for selecting the augmentation technique you use? (Multiple answers possible)**

- Personal experience/expertise

- Patient preference

- Economic reasons

- Referrer’s request

- Practice and organizational structure

- Preoperative time expenditure (planning, preparation, etc.)

- Intraoperative time expenditure (duration of surgery, etc.)

- Potential complication risk

- Evidence/literature

- Costs

**What factors do you consider when selecting the type of bone or bone substitute material (BSM)? (Multiple answers possible)**

- Personal experience/expertise

- Individual situation and indication

- Patient preference

- Referrer’s request

- Practice and organizational structure

- Preoperative time expenditure (planning, preparation, etc.)

- Intraoperative time expenditure (duration of surgery, etc.)

- Handling during the procedure

- Stability/resorption time of the BSM

- Potential complication risk

- Evidence/literature

- Costs

**What factors do you consider when selecting a membrane? (Multiple answers possible)**

- Personal experience/expertise

- Individual situation and indication

- Patient preference

- Referrer’s request

- Practice and organizational structure

- Preoperative time expenditure (planning, preparation, etc.)

- Intraoperative time expenditure (duration of surgery, etc.)

- Handling during the procedure

- Stability/resorption time of the membrane

- Potential complication risk

- Evidence/literature

- Costs

- Biological safety

**What preoperative management do you use before or during augmentation procedures? (Multiple answers possible)**

- Preemptive analgesia (e.g., ibuprofen)

- Glucocorticoids (e.g., dexamethasone)

- Oral antibiotics

- Intravenous antibiotics

- CHX rinsing

- Professional dental cleaning

- Optimization of patient compliance

- Optimization of oral hygiene

- Smoking cessation

**What postoperative management do you use after augmentation procedures? (Multiple answers possible)**

- Pain medication as needed

- Pain medication according to a fixed schedule

- Oral antibiotics

- Intravenous antibiotics

- CHX rinsing

- Cooling

- (Kinesio) taping

- Bromelain

- Arnica

- Prohibition of sports/physical activity

- Smoking cessation

- Adjustment of mucosa-supported prostheses in the augmentation area

- Soft relining of mucosa-supported prostheses in the augmentation area

- Prothesis avoidance

**What complications do you generally observe after augmentation procedures? (Multiple answers possible)**

- Infections

- Abscesses

- Postoperative bleeding

- Membrane exposure

- Dehiscence

- Wound healing disturbances

- Insufficient augmentation volume over time

- Augment loss

**What factors do you believe influence the long-term success of augmentations? (Multiple answers possible)**

- Quality of the materials used

- Surgical technique

- Experience/expertise of the operator

- Patient health/compliance

- Oral hygiene

- Medications and comorbidities of the patient

- Smoking status

- Aftercare

**What imaging do you prefer after augmentation procedures? (Multiple answers possible)**

- Dental film

- Panoramic radiograph

- CBCT

- CT

- Intraoral scanner

- Face scanner

- No imaging

**What do you consider relative contraindications for augmentation procedures? (Multiple answers possible)**

- Well-controlled diabetes mellitus

- Poorly controlled diabetes mellitus

- History of radiation in the augmentation area within the last year

- History of radiation in the augmentation area, not within the last year

- Use of proton pump inhibitors

- Antiresorptive drugs for benign conditions

- High-potency antiresorptive drugs for malignant conditions

- Smoking

- Poor oral hygiene

- Poor compliance

- Mucosa-supported prostheses in the augmentation area

**What do you consider absolute contraindications for augmentation procedures? (Multiple answers possible)**

- Well-controlled diabetes mellitus

- Poorly controlled diabetes mellitus

- History of radiation in the augmentation area within the last year

- History of radiation in the augmentation area, not within the last year

- Use of proton pump inhibitors

- Antiresorptive drugs for benign conditions

- High-potency antiresorptive drugs for malignant conditions

- Smoking

- Poor oral hygiene

- Poor compliance

- Mucosa-supported prostheses in the augmentation area

**Do you utilize vitamin D level determination for patients before augmentation procedures?**

- YES, performed in the practice

- YES, performed via an external laboratory after blood collection

- NO

**Which innovative developments do you find most promising for augmentation? (Multiple answers possible)**

- Bioprinting of bone and bone substitute materials (BSM)

- Use of stem cells

- Intraoperative navigation

- Advances in CAD/CAM technology

- New synthetic biomaterials

- Innovative resorbable biomaterials (e.g., magnesium screws)

- None of the above

**What challenges do you see in applying biomaterials (BSM and membranes) in practice? (Multiple answers possible)**

- Costs

- Time expenditure

- Storage requirements

- Personnel resources

- Availability

- Handling

- Documentation

- Long-term stability of the augmentation

- Behavior in the event of peri-implantitis

- Biological safety (cell residues, bacteria, viruses, prions, etc.)

- Patient acceptance

- Market overview and product diversity

**How do you inform yourself about biomaterials? (Multiple answers possible)**

- Professional journals

- Scientific literature

- Internet

- Continuing education

- Conferences

- Colleagues

- Representatives

**Which general statements about augmentations do you agree with? (Multiple answers possible)**

- I avoid them whenever possible

- Prefer shorter and narrower implants over augmentation when possible

- As little as possible, as much as necessary

- Tend to augment generously and preventively if necessary

- Autologous bone and soft tissue are the gold standard for me

- Biomaterials are a good alternative to autologous transplants

**What importance does the skeletal envelope (also known as the bony envelope) have for you in augmentations? (Multiple answers possible)**

- I try to augment within the skeletal envelope

- I also augment outside the skeletal envelope

- I augment situationally, both within and outside the skeletal envelope

- I do not consider the skeletal envelope in augmentations

- The skeletal envelope has no significance to me

**What suture materials do you prefer for augmentations? (Multiple answers possible)**

- Resorbable monofilament

- Resorbable braided

- Non-resorbable monofilament

- Non-resorbable braided

- Tissue adhesive (e.g., cyanoacrylate)

**What methods of biologizing biomaterials (BSM and membranes) do you use? (Multiple answers possible)**

- Autologous blood

- Platelet-rich fibrin (PRF)

- Platelet-rich plasma (PRP)

- Growth factors (e.g., BMP-2)

- Enamel matrix proteins (e.g., Emdogain®)

- None
