# Supplementary material for: Alveolar ridge augmentation in Oral and Maxillofacial Surgery: a study on current practices, patient management and innovations in Germany
Source: Int J Implant Dent. 2025 Apr 16;11:31. doi: 10.1186/s40729-025-00619-5 (PMC12003255; doi:10.1186/s40729-025-00619-5)
Supplement: Supplementary file 2 — Supplementary material 2. Corresponding data to figures 1–6. [file 40729_2025_619_MOESM2_ESM.docx]

| Imaging Modality | Percentage (%) | Number of Responses |
| --- | --- | --- |
| Dental X-Ray Film | 5.97% | 19 |
| Panoramic X-Ray | 63.84% | 203 |
| CBCT (Cone Beam CT) | 87.74% | 279 |
| Computed Tomography (CT) | 4.72% | 15 |
| Intraoral Scanner | 17.92% | 57 |
| Face Scanner | 0.94% | 3 |
| No Imaging | 0.00% | 0 |
| Total Participants | - | 318 |

Corresponding data to Figure 1.

| Procedure/Technique | Percentage (%) | Number of Responses |
| --- | --- | --- |
| Ridge Preservation | 74.75% | 222 |
| Internal Sinus Lift | 90.57% | 269 |
| External Sinus Lift | 97.31% | 289 |
| GBR with Resorbable Membranes | 83.16% | 247 |
| GBR with Non-Resorbable Membranes | 29.97% | 89 |
| Umbrella Technique | 34.01% | 101 |
| Shell Technique | 57.91% | 172 |
| Bone Block Augmentation | 73.40% | 218 |
| Ridge Split | 46.46% | 138 |
| Vertical Sandwich Osteotomy with Interpositional Graft | 17.85% | 53 |
| Pre-fabricated Titanium Mesh | 14.81% | 44 |
| Patient-Specific CAD/CAM Titanium Mesh | 21.89% | 65 |
| Patient-Specific CAD/CAM Bone Block | 12.12% | 36 |
| Distraction | 13.47% | 40 |
| Bone Ring | 8.75% | 26 |
| Total Participants | - | 297 |

Corresponding data to Figure 2.

| Membrane Type | Percentage (%) | Number of Responses |
| --- | --- | --- |
| Collagen Membrane | 83.84% | 249 |
| PTFE Membrane | 6.06% | 18 |
| Titanium-Reinforced PTFE Membrane | 13.13% | 39 |
| Magnesium Membrane | 2.02% | 6 |
| PRF Membrane | 35.02% | 104 |
| Other Non-Resorbable Membrane | 3.03% | 9 |
| Other Resorbable Membrane | 14.14% | 42 |
| Total Participants | - | 297 |

Corresponding data to Figure 3.

| Criteria | Percentage (%) | Number of Responses |
| --- | --- | --- |
| Personal Experience/Expertise | 95.59% | 260 |
| Individual Situation and Indication | 75.00% | 204 |
| Patient Preference | 42.65% | 116 |
| Referrer’s Request | 5.88% | 16 |
| Practice and Organizational Structure | 10.66% | 29 |
| Preoperative Time Effort (Planning, Preparation, etc.) | 8.82% | 24 |
| Intraoperative Time Effort (Surgery Duration, etc.) | 22.79% | 62 |
| Intraoperative Handling | 47.79% | 130 |
| Longevity/Resorbability of the Graft Material | 37.87% | 103 |
| Potential Complication Risk | 55.88% | 152 |
| Evidence/Literature | 54.78% | 149 |
| Costs | 30.51% | 83 |
| Total Participants | - | 272 |

Corresponding data to Figure 4.

| Criteria | Percentage (%) | Number of Responses |
| --- | --- | --- |
| Personal Experience/Expertise | 95.59% | 260 |
| Individual Situation and Indication | 68.75% | 187 |
| Patient Preference | 22.79% | 62 |
| Referrer’s Request | 4.04% | 11 |
| Practice and Organizational Structure | 9.56% | 26 |
| Preoperative Time Effort (Planning, Preparation, etc.) | 5.88% | 16 |
| Intraoperative Time Effort (Surgery Duration, etc.) | 18.01% | 49 |
| Intraoperative Handling | 46.69% | 127 |
| Longevity/Resorbability of the Membrane | 45.96% | 125 |
| Potential Complication Risk | 41.91% | 114 |
| Evidence/Literature | 48.53% | 132 |
| Costs | 31.99% | 87 |
| Biological Safety | 37.13% | 101 |
| Total Participants | - | 272 |

Corresponding data to Figure 5.

| Imaging Modality | Percentage (%) | Number of Responses |
| --- | --- | --- |
| Dental X-Ray Film | 15.07% | 41 |
| Panoramic X-Ray | 87.50% | 238 |
| CBCT (Cone Beam CT) | 45.59% | 124 |
| Computed Tomography (CT) | 0.74% | 2 |
| Intraoral Scanner | 3.68% | 10 |
| Face Scanner | 0.37% | 1 |
| No Imaging | 1.10% | 3 |
| Total Participants | - | 272 |

Corresponding data to Figure 6.
